# Molecular Crystallization Inhibitors for Salt Damage Control in Porous Materials: An Overview

**DOI:** 10.3390/molecules25081873

**Published:** 2020-04-18

**Authors:** Maria Paola Bracciale, Svetlana Sammut, JoAnn Cassar, Maria Laura Santarelli, Assunta Marrocchi

**Affiliations:** 1 Department of Chemical Engineering Materials and Environment and CISTeC- Research Center in Science and Technology for the Conservation of Historical-Architectural Heritage, Sapienza University of Rome, Via Eudossiana 18, 00184 Rome, Italy; mariapaola.bracciale@uniroma1.it (M.P.B.); marialaura.santarelli@uniroma1.it (M.L.S.); 2 Department of Conservation and Built Heritage, Faculty for the Built Environment, University of Malta, Msida MSD 2080, Malta; svetlana.sammut.00@um.edu.mt (S.S.); joann.cassar@um.edu.mt (J.C.); 3 Department of Chemistry, Biology and Biotechnology University of Perugia, Via Elce di Sotto 8, 06123 Perugia, Italy

**Keywords:** crystallization inhibitors, sustainability, salt decay, porous materials, built heritage

## Abstract

The use of inhibition chemicals holds the prospect of an efficient strategy to control crystallization in porous materials, thereby potentially contributing to the prevention or mitigation of the salt decay phenomenon in modern as well as historical building materials in a more sustainable manner. In this review, we first provide an essential background on the mechanism of salt crystallization and on the factors influencing this phenomenon; next, we illustrate the mechanism at the basis of the action of crystal growth inhibitors, and critically discuss the major advances in the development of different families of inhibitors, particularly focusing on their influence on salt transport and crystallization within the structure of porous media. Specifically, correlations between the crystallization inhibition processes in porous materials and variables, such as porous substrate composition and properties, contaminant salt type and concentrations, microclimatic conditions, inhibiting solution concentration and properties, and application methods, will be highlighted. Environmental aspects, limitations, and problems associated with some inhibition chemicals are also taken into account. Finally, a survey and a discussion on the most representative experimental techniques and instrumentation available to assess qualitatively and quantitatively the inhibitor effectiveness, as well as recently developed modelling tools are given out.

## 1. Introduction

**Figure 1:**
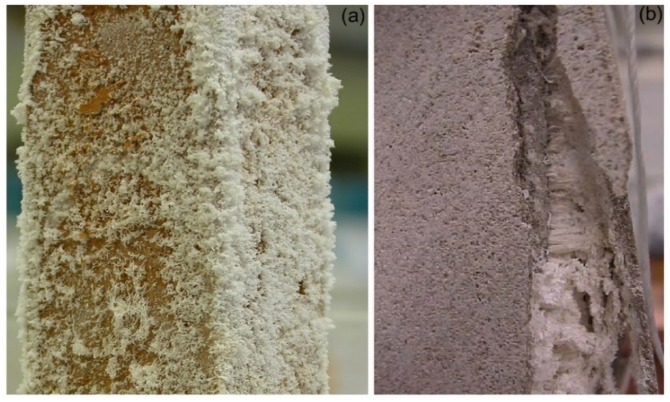
Two typical salt-decay patterns: (**a**) Efflorescence and damage to a brick caused by sodium sulfate crystallization; (**b**) Limestone block showing surface detachments due to subflorescence phenomena, induced by sodium sulfate.

**Figure 2:**
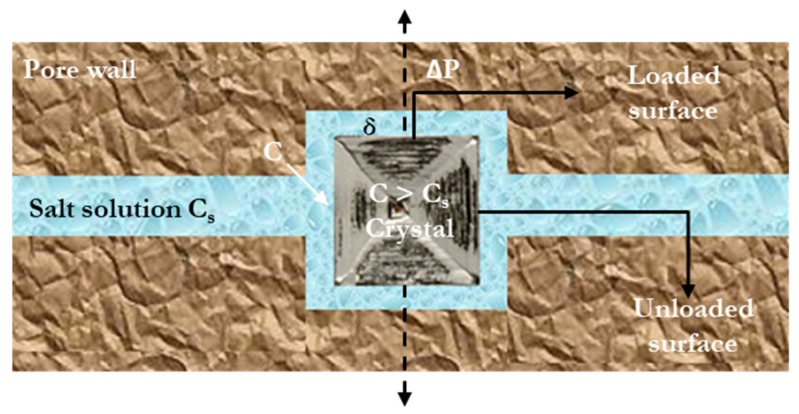
Schematic representation of a crystal confined between the pore walls in the presence of a salt solution layer (*δ*). *C* is the solute concentration in the supersaturated solution; *C_s_* is the solute concentration in the saturated solution.

Salt damage is commonly recognized as a major cause of decay for porous construction materials, including concrete, mortar, limestone, sandstone, and bricks, and is especially relevant in historical buildings as well as archaeological sites [1,2,3,4,5,6,7,8]. Salt decay is generally understood as a temperature/humidity-dependent weathering process associated with (1) the presence of salt crystals on the surface of porous materials (i.e., efflorescence), which is often highly visible and impressive, but generally results in little damage (Figure 1a); (2) the mechanical stresses introduced by salt crystals deposited within the material pores (i.e., subflorescence or cryptoflorescence), which may ultimately endanger the structural integrity of the materials (Figure 1b) as well as cause widespread loss of surface, e.g., exfoliation, detachments (vide infra).

Due to the large number of heritage sites all over the world affected by salt decay, as well as the harmfulness and complexity of such phenomena, multidisciplinary research in built heritage conservation, with particular reference to the behavior of materials, conservation practices, and possible environmental effects, have acquired great importance. Despite the impressive achievements in the understanding of decay mechanisms, several fundamental issues have not yet been fully understood. Consequently, adequate prevention or mitigation strategies are often lacking, particularly because it is generally not feasible to eliminate the source of salts and/or to control the microclimatic environment.

To date, water washing followed by the application of surface-active poulticing using highly absorbent materials, such as clays or paper pulp, is among the most common conservation treatment methodologies for salt decay [9,10,11,12]. Other methods include chemical cleaning by, e.g., hydrofluoric acid, salt extraction by barium compounds, electromigration, convection (vacuum or water pressure extraction), the creation of chemical barriers, and the employment of a variety of consolidants and surface coatings, such as water repellents, i.e., polymers passivating the mineral surface [13]. However, such techniques are sometimes ineffective or too expensive, therefore justifying the need to find new approaches.

Over the past few years, there has been ever-growing attention towards the development of crystallization inhibition chemicals as an innovative strategy for controlling salt crystallization in porous materials. This approach offers the prospect of a more sustainable conservation of the built heritage, as well as the protection of modern buildings [14,15]. The field of sustainability is becoming crucial, as a result of the ever more stringent regulatory requirements in the European Union, North America, and developed Asian countries [16].

Generally speaking, the important effect of additives on the growth of salt crystals has been known since the 18th century [17,18]. Such additives, which can be ions or molecules, alter the surface properties of the crystals; this leads to changes in nucleation, growth, and thereby to changes in the shape of the crystals as well as in their agglomeration/dispersion behavior. Examples of additives with extensive technological and industrial uses [19,20,21,22] are the families of (poly)phosphates [23,24,25,26], (poly)carboxylates [27,28], and benzotriazoles [29]. These additives are well-known scale inhibitors, preventing the undesired effects associated with sparingly soluble salts (e.g., sulfates, carbonates) precipitating in oil extraction pipelines [30], industrial boilers, heat exchangers, house appliances or water pipes [25,29], and others.

Although the effectiveness of the abovementioned salt crystallization inhibitors in bulk solution has largely been proven [19,20,21,22,23,24,25,26,27,28,29,30], extensive experimental studies are still needed to identify the benefits and risks of using these products for the prevention of salt decay in building materials, where the action is limited inside the pores of the material.

The main purpose of this review is to give a critical view of the most representative advances in the development of crystal growth inhibitors to halt/mitigate salt damage in porous building materials. We will first briefly discuss the salt deterioration of porous media from a theoretical point of view, to provide an essential background for the subsequent sections. Next, we will focus on the key families of inhibition chemicals (i.e., biomass-derived chemicals, phosphonates, alkali ferrocyanides, surfactants) and their influence on salt transport/crystallization within the structure of porous materials. Limitations and problems associated with such inhibition chemicals, along with the most representative instrumentation/techniques available to assess their effectiveness, will be also illustrated and discussed.

## 2. Fundamental Mechanisms and Influencing Factors for Salt Crystallization Damage

Salt decay occurs by the concomitant presence of soluble salts and water in a porous material, at given environmental conditions and ensuing crystallization/dissolution cycles. The phenomenon originates from salt ions (vide infra) that migrate while dissolved in liquid water, which flows in the pore network of building materials, and then the subsequent evaporation of the water.

Liquid water may penetrate such materials by different processes, including hygroscopic moisture, penetration of rainwater through, e.g., infiltration through the porous material itself, construction joints, damaged roofs and cracks, dew-point condensation, and rising damp. The latter is probably the most frequent source of water (and salt) ingress, and perhaps one of the most difficult to eliminate, particularly when dealing with old buildings.

Common soluble salts in porous materials [4] are different types of chlorides, sulfates, nitrates, and carbonates, each with a different solubility, crystalline structure, and crystallization parameters. Chlorides, for instance, often arise from direct contact of building materials with seawater by marine fog, seawater spray, or contaminated groundwater or de-icing salts. Chlorides may also come from salt-infested items, once stored in the building. Nitrates may be introduced by animal excrements or by microbiological activity. Sulfates can originate from ceramic materials or pollution. The gypsum (Ca_2_SO_4_·2H_2_O) contained in many plasters, or even repairs, may dissolve and migrate into other adjacent porous building materials. Carbonates typically originate from high alkali content materials, e.g., cement-based mortars. Indeed, the alkali hydroxides in these materials react with carbonic acid (which results from, e.g., dissolution of aerial CO_2_ in the water in damp walls) and thereby form alkali carbonates; they are also the basic component of limestones, some sandstones, and lime-based mortars and plasters. These are the most common sources, although many other salt types and salt origins are possible in old buildings.

In brief, the mechanism of salt crystallization from a solution initiates via the aggregation of the initially dispersed (salt) solute ions; this nucleation eventually leads to the formation of clusters and finally crystals [31] depending on (1) the existing conditions in terms of temperature, relative humidity, type of ions in solution, and eventual impurities; and (2) on the type of intrinsic porous system, which affects water transport.

The driving force for crystallization from solution (i.e., nucleation, precipitation, growth, etc.) is the difference between the chemical potential of free ions in solution and nuclei, which is directly related to supersaturation of the salt in solution, i.e., the ratio between the ion activity product in solution and the solubility constant of the salt [18].

It is generally accepted that material damage occurs when the tensile stress caused by the crystals growing in the confined space of the porous matrix exerts a pressure that is greater than the tensile strength of the porous material [32,33,34,35,36].

The ability of salt to induce damage relies more specifically on the presence (at least temporary) of a supersaturated solution, and ultimately a layer (*δ*), between the pore walls and the crystal formation, which enables the diffusion of ions contributing to the continued growing crystal surfaces [32,33,34,35,36] (Figure 2). The solution layer is provided by the action of repulsive forces, or “disjoining pressure”, between the growing salt crystal and the pore wall. The higher the supersaturation ratio of the solution layer, the higher the pressure exerted by the growing crystal. This was first expressed by Correns’ equation (Equation (1)) [32]:

(1) $$\Delta P=P_{cr}-P_{l}=\left(\frac{RT}{V_{m}}\right)\ln\left(\frac{C}{C_{s}}\right),$$

to derive which, the growth of a pre-existing crystal confined between two plates under a load and immersed in a salt solution was considered.

Here, Δ*P* (MPa) is the crystallization pressure, *P_cr_* (MPa) is the pressure on the loaded face of the growing crystal, *P_l_* is the hydrostatic (ambient) pressure of the pore solution, *R* (8.3145 MPacm^3^mol^−1^k^−1^) is the gas constant, *T* (°K) is the absolute temperature, *V_m_* (cm^3^mol^−1^) is the molar volume of the solid phase, *C_s_* is the solute concentration in a saturated solution, and *C* is the solute concentration in the supersaturated solution. Importantly, any alteration of the conditions bringing the crystals in contact with the pore walls, thus eliminating the solution film, could stop their growth and, consequently, no crystallization pressure would be exerted [31,33,35,37].

Following the earliest theories postulated by Correns [32], various approaches by different authors have been investigated in the literature to calculate the crystallization pressure [34,38,39,40,41,42,43], including that of using solute activities of solutions instead of concentrations [44], and giving the maximum stress as a function of the crystal curvature [45]. Particularly, Steiger [34] correlated the chemical potential of each face (loaded and unloaded) of a crystal confined in a porous materials to the pore solution, depending on the orientation of the crystal faces relative to the pore wall. The non-ideal behavior of the liquid phase was also taken into account [34,43], along with the temperature dependence of the interacting parameters involved. Steiger, ultimately, derived an equation (Equation (2)) for calculating the crystallization pressure generated by a regularly shaped crystal, any size, in a pore, when in contact with a supersaturated solution, by considering a combined realistic approach that includes both the degree of supersaturation of the solution and the effect of the curvature of the crystal–liquid interface, as follows:

(2) $$\Delta P=\left(\frac{RT}{V_{m}}\right)\ln\left(\frac{a}{a_{\infty }}\right)-\gamma_{el}\left(\frac{dA}{dV}\right),$$

where *R* is the gas constant, *T* is the absolute temperature, *V_m_* is the molar volume of the solid phase, *a* is the salt activity in the supersaturated solution, *a_∞_* is the equilibrium activity of a bulk crystal under hydrostatic (ambient) pressure, and *V*, *A*, and *γ_el_* are the volume, the surface, and the interfacial free energy of the crystal, respectively [34]. In the case of irregularly shaped crystals in pores, Equation (2) was reported to be applicable to each individual crystal face [34]. It must be noted that, besides the crystallization pressure, possible damaging mechanisms are the crystal hydration pressure and the crystal volumetric variation associated with multiple dissolution and precipitation steps, as it happens with daily (micro)climate changes (the so-called crystallization cycles) [46].

As already mentioned in the introduction section, the occurrence of material damage and its patterns depends largely on the location where salts crystallize. Indeed, in evaporative crystallization processes, crystals precipitate at the drying front where the water evaporates and hence, here, the solution concentration increases up to (super)saturation. Efflorescence takes place when the liquid flux within the pores is high enough to compensate for the evaporative demand and, therefore, is able to reach and evaporate on the outer surface of the material. Subflorescence (or cryptoflorescence) occurs when the liquid flux is lower than this and, hence, the drying front is located inside the material. This drying, and consequently, progressive slowing and loss of solution transport within the pores, is generally accompanied by a reduction in temperature as water evaporates and supersaturation increases [31,37].

It is worth mentioning that in-pore crystallization causes a reduction of the material pore volume, which may hinder solution transport, generating so-called pore clogging. Since this affects the location/quantity of crystals, in most cases, it may lead to progressive stress development and deterioration of the material [47].

Evaporative drying of porous media and, ultimately, the occurrence of efflorescence or subflorescence (cryptoflorescence), are generally recognized as being jointly controlled by media internal transport properties and external (atmospheric) conditions, e.g., air temperature/velocity, relative humidity (RH), and solar radiation. In particular, the rate of capillary transport in porous materials may differ for diverse solutions, owing to differences in these solutions in terms of viscosity, surface tension, and/or contact angle with the material [2,3,47,48,49,50,51,52,53,54,55]. For instance, Rodriguez-Navarro and Doehne [3] reported that depending on a given type of salt and solution concentration, capillary flow can be markedly slow for some salt solutions, as demonstrated for sodium sulfate (Na_2_SO_4_) in comparison to sodium chloride (NaCl). Therefore, these solutions barely reach the drying surface, thereby having a great tendency to form harmful subflorescence. Regarding the influence of environmental conditions, temperature-induced crystallization may occur for salts whose solubility is temperature dependent; this is exemplified by Na_2_SO_4_. Indeed, the stable phase in contact with a saturated solution of Na_2_SO_4_ is mirabilite (Na_2_SO_4_∙10H_2_O) or thenardite (anhydrous Na_2_SO_4_) depending on whether the temperature is below or above 32.4 °C, respectively. The heptahydrate (Na_2_SO_4_∙7H_2_O) may also exist below 32.4 °C, in contact with a saturated solution; however, this is not an equilibrium form, i.e., the solution is supersaturated with respect to mirabilite [51]. The great deteriorating effects caused by this characteristic have been reported by many authors [48,51,52,53,54,55].

Schaffer [56] observed that salt crystallization may cause greater damage to stones with small pores than to those with larger pores. Moreover, Rodriguez-Navarro and Doehne [3] claimed that salt solutions are drawn from the larger to the smaller pores during drying. Thus, according to this theory, crystallization occurs in the smaller pores, whereas the larger pores act as solution reservoirs. However, in 2004, Scherer [57] argued that crystallization may take place both in small and in large pores. Particularly, he reported that “*under equilibrium conditions, where the crystal is surrounded by a film of solution, high stresses are expected only in small pores, but when that film is discontinuous (as may occur during drying) high stresses can arise also in large pores*”. Salt crystallization in large pores (i.e., pores of diameter from 1 to 10 nm) was in fact observed by Zehnder and Arnold [58] within laboratory tests on brick and mortar samples.

It has been demonstrated that the material’s porosity and pore size distribution also influence the decay process at a macroscopic level [3,59,60]. For instance, Rodriguez-Navarro and Doehne [3] argued that small-pored stones are more susceptible to increased salt decay, because a different hydric flux balance arises during drying; particularly, (a) liquid migration is slower in smaller than in larger pores; and (b) smaller pores result in a larger surface area, i.e., the liquid evaporation rate is higher. Subflorescence may therefore occur more easily in small-pored stones. Further, it is worth noting here that a high evaporation rate can also lead to higher supersaturations; that is, to stronger crystallization pressures [2,3,11,49,50,61,62].

Finally, the specific habit that the salt crystal assumes (e.g., prismatic, dendritic, needle-like or cubic) is also dependent on many factors [37,58,63,64,65,66], including the salt type, porosity, and pore sizes of the substrate; solution properties; nucleation and crystal growth kinetics; as well as environmental conditions, and substrate humidity. Chatterjee, in particular [67], argued that some crystalline habits seem to be associated with more severe damage in porous materials when in-pore crystallization occurs [37] because of a greater tensile stress, which may be, in turn, attributed to the different surface contact between the crystal and the pore wall [1].

## 3. Crystal Growth Inhibitors

### 3.1. Mechanisms of Action

**Figure 3:**
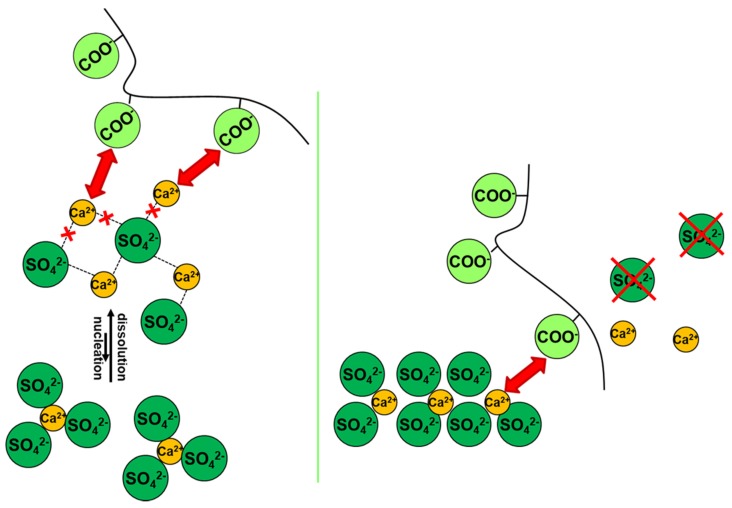
Cartoon picture showing a possible inhibitor acting by limiting the formation of stable nuclei (left panel) and by modifying the crystalline habitus (right panel) of a salt, exemplified by Ca_2_SO_4_. Dashed lines in the left panel are exemplificative for the interaction occurring between the ions in solution, leading to salt that nucleates. The red arrows in both panels represent the interaction between ions and the CO_2_^−^ functional groups of the model inhibitor.

Inhibitors are considered to act according to two main mechanisms [20,21,68,69,70], i.e., by (a) preventing or delaying the formation of stable nuclei, thus keeping salt precipitation from occurring and/or (b) modifying the crystalline habit by adsorption on specific faces of a growing crystal, thus decreasing its growth rate. Importantly, inhibitors are primarily designed to form enough coordinative bonds with the ions of the mineral surface [20,21,68,69,70] (Figure 3); this allows them to be active even in traces.

It is generally accepted that the preferential adsorption of the inhibitors on a growing crystal surface is an essential step in the specific action of the species, though the role of the factors influencing such step is not yet fully understood.

**Figure 4:**
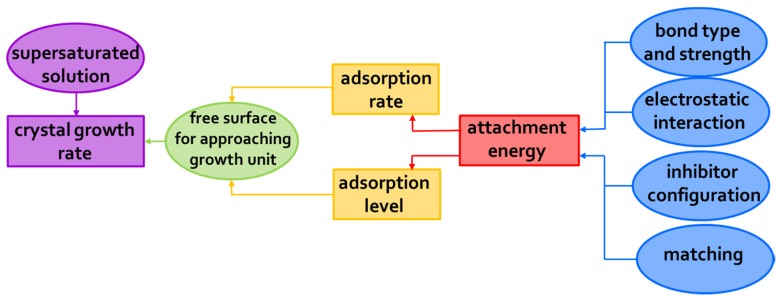
Factors influencing the inhibitor adsorption process on a crystal surface.

In this regard, however, it is now accepted that regular crystal growth kinetics is somewhat perturbed by the adsorption of inhibitor molecules, which compete with the normal growth units. The extent of such perturbation is determined by the inhibitor adsorption level and the adsorption rate [69] (Figure 4). These factors depend, in turn, on the so-called attachment energy, which is influenced by several parameters, including those identified by van der Leeden and van Rosmalen [69], i.e., the “*type and strength of the bonds between surface ions and functional groups of the inhibitor, their electrostatic interaction, the inhibitor configuration, and the matching of the interatomic distances/orientation of the lattice ions in the crystal surface layer with respect to the functional groups of the inhibitor adsorbed on the crystal surface*”. More precisely, the adsorption level/adsorption rate are determined by the inhibitor affinity towards the crystal surface. The attraction forces can be of the electrostatic type and, to make them effective, at least some ionization of the functional groups (typically acidic) of the inhibitor is needed, to enable hydrogen and/or coordinative bonds to form with the ions at the crystal surface. On the other hand, total ionization of the inhibitor may prevent it from being adsorbed, since the gain in energy due to the bonding with the crystal surface is not sufficient to overcome the associated entropy loss [20,21,68,69,70]. The surface coverage needed to obtain effective crystal growth inhibition then depends on the type of inhibitor, the pH-value of the microenvironment, the type of salt, and its surface relief related to its mechanism of growth [51,71,72,73]. For instance, in 2006, Ruiz-Agudo et al. [51] demonstrated the influence of pH (i.e., degree of the deprotonation of the functional groups) on the inhibition effect of three phosphonates, i.e., 1-hydroxyethylidene-1,1-diphosphonic acid (C_2_H_8_O_7_P_2_; HEDP), aminotris(methylenephosphonic acid (C_3_H_12_NO_9_P_3_; ATMP), and diethylenetriaminepentakis-(methylphosphonic acid) (C_9_H_28_N_3_O_15_P_5_; DTPMP), on sodium sulfate (Na_2_SO_4_) crystallization. Particularly, they found that an increase in pH increased the inhibition effectiveness of such phosphonates on Na_2_SO_4_ precipitation up to pH~8. At higher pH, the degree of inhibition was demonstrated to become less, thereby indicating that a higher number of phosphonate groups does not guarantee that a molecule will have a greater inhibition capacity.

Additionally, a “planar” configuration of the inhibitor upon a crystal surface may promote an effective exploitation of its attraction as well as bonding abilities (“inhibitor configuration” factor in Figure 4). Note that a possible design strategy to achieve this goal is avoiding the introduction of hydrophobic structural features and/or non-functional tails [68,69,70]; in fact, these may both extend out from the crystal surface and cause [68,69,70] steric hindrance by, e.g., hampering the access of the inhibitor’s functional groups to the crystal surface, which ultimately results in a low adsorption power. Finally, appropriate matching between the inhibitor functional groups and, e.g., the cations at the crystal surface may facilitate the adsorption process (“matching” factor in Figure 4) [74]. The matching process is dependent on interatomic distances as well as the orientation of structural units in the crystal surface layer and charge distribution.

Taking all these considerations into account, it is clear that a precise molecular design is therefore needed for inhibitors to be effective.

### 3.2. Inhibition Chemicals for Porous Media Applications

In this section, we will focus on the different families of inhibition chemicals, which stand as the most representative to date; their ability to modify the generally destructive interaction between salts, water, and porous materials will be discussed (Table 1).

#### 3.2.1. Biomass-Derived Species

In recent years, our group has undertaken broad research [49,75,76,77,78,79,80,81] focusing on the effect of biomass-derived inhibitors on saline solutions percolating through and crystallizing in porous media after water evaporation, in order to develop a sound methodology suitable for addressing the conservation needs of different salt-weathered sites. Our attention has been particularly directed towards the crystallization inhibition properties of so-called platform polycarboxylate molecules (i.e., maleate (C_4_H_4_O_4_), citrate (C_6_H_8_O_7_), tartrate (C_4_H_6_O_6_)) [82] and their phosphorylated derivatives.

As mentioned above, the inhibitory efficiency depends on the presence of acidic groups or negatively charged ionic species. On this basis, compounds possessing up to five possible dissociation groups have appeared to us as worthy of consideration. Notably, all the investigated inhibitor systems, besides being derived from a renewable source, are non-toxic, and exhibit complete solubility in water and/or alcohol, thereby avoiding the use of harmful organic solvents in their production. These features enable the use of inhibitors in accordance with the volatile organic compounds (VOCs) emission control and safety directives during the conservation procedures. Furthermore, there is the advantage that the investigated compounds, from the point of view of degradation products, totally meet environmental requirements.

**Figure 5:**
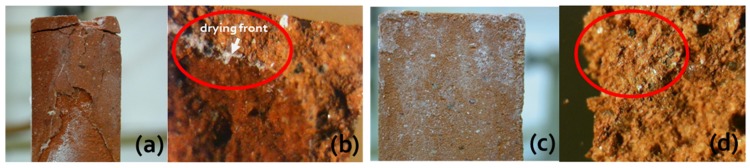
Brick sample showing surface deteriorations due to subflorescence, induced by Na_2_SO_4_ in (**a**) the absence and (**c**) the presence of phosphocitrate PC inhibitor. Micrographs showing Na_2_SO_4_ crystals (**b**) strongly localized below the material surface in the absence of PC and (**d**) the distribution in the presence of PC inhibitor.

In a series of papers [49,75,76,77,78,79,80,81], we demonstrated that phosphocitrate (C_6_H_9_O_10_P; PC) [83,84] is one of the most promising and versatile inhibitors, because of its effectiveness in controlling the crystallization of different salts (e.g., sodium sulfate (Na_2_SO_4_), sodium chloride (NaCl), sodium nitrate (NaNO_3_), calcium carbonate (CaCO_3_), and their binary/ternary mixtures) in a wide range of porous materials and under various relative humidity (RH)/temperature (T) conditions. As for the materials, we focused on brick, tuff, and different types of limestone. PC was selected based on previous research, which has focused on its ability to control the deposition of calcium phosphates and oxalates either under in vitro-controlled media conditions or in vivo associated with simulated pathological states [21,85,86,87,88,89]. More recently, Sallis and co-workers demonstrated its ability to control calcite (CaCO_3_) and gypsum (CaSO_4_·2H_2_O) formation and deposition in solution [20,21,90]. Besides, PC was also found to influence the crystallization of two well-known magnesium crystallites, i.e., magnesium ammonium phosphate (NH_4_MgPO_4_·6H_2_O; struvite) and magnesium hydrogen phosphate (MgHPO_4_·3H_2_O; newberite) [91]. We observed that, in most cases, the presence of PC favors the flux of saline solution through a porous material, i.e., the inhibitor helps crystallization to occur on the surface of materials and not within the pores, thereby leading to negligible subflorescence and, ultimately, markedly limiting the materials’ structural damage. This is exemplified in Figure 5a,c for the case of the phosphocitrate/brick/Na_2_SO_4_ system.

Such a phenomenon may be ascribed to the abovementioned inhibiting role of phosphocitrate on salt nucleation, which prevents the salt from crystallizing within the material pores, thus inducing crystallization where a very high supersaturation occurs, i.e., on the material surface, where the evaporation is at a maximum (see Section 2). Again, with reference to the above case, these findings were corroborated by digital microscope observations, which revealed that salt crystals were far more clearly localized below the surface of the reference samples without compared to with the presence of PC (Figure 5b,d).

Furthermore, it was found that phosphocitrate enabled a variety of salts/salt mixtures to crystallize as thin filament-like efflorescence, and not as a thick crust, thus preventing any marked aesthetic impact, which might compromise the visual integrity of the material. It was hypothesized that the inhibitor delayed the initiation of crystallization, which occurred at higher supersaturation than in the absence of the inhibitor; this in turn affected the crystal morphology. In fact, it is known (vide supra) that when crystallization takes place at a high supersaturation, the crystal will have a morphology very different from the equilibrium crystal shape.

**Figure 6:**
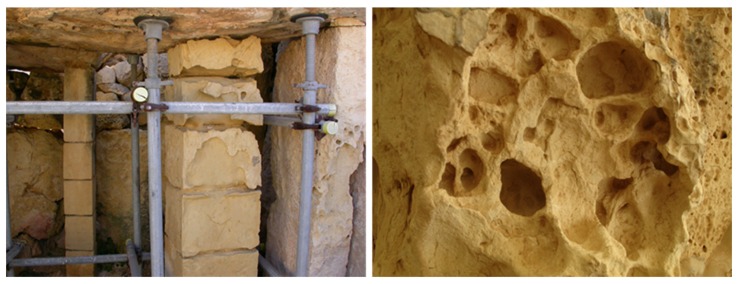
Example of back-weathering (left) and alveolar weathering (right) in globigerina limestone.

For instance, PC was tested on the main types of soft, porous globigerina limestone [78], i.e., franka (bajda and safra) and soll, using freshly quarried samples, to control sodium sulfate (Na_2_SO_4_) crystal growth. For comparison purposes, crystallization experiments employing a PC non-phosphorylated counterpart, i.e., citrate (C_6_H_8_O_7_), were performed. Note here that globigerina limestone is the main building stone in the Maltese Islands, of which the lower member is the commonly used. This occurs in two main types as representative of a continuum of “types”, as recognized by the local construction industry and determined by porosimetric tests, the more durable franka and the more easily weathered soll [50,92,93,94]. Weathering of this stone can often be seen locally in buildings of all ages/archaeological sites as a varying degree of alveolar (or honeycomb) weathering, powdering, and flaking, often with a loss of much of the stone thickness (back-weathering) in the process (Figure 6). This can be attributed to the interaction of the intrinsic properties with the external conditions, as is usually the case; in this case, the environment is particularly aggressive with respect to marine salt and accompanying high humidity. The study was carried out in conditions where an Na_2_SO_4_ saline solution percolated through, and evaporated from, saturated porous media specimens. The latter were previously wet with aqueous solutions of the inhibitors at concentrations ranging from 1 to 10 ppm, then slowly dried in a controlled environment (25 ± 2 °C, 50 ± 5% relative humidity). As a reference, blank samples (with no inhibitors) for each type of globigerina limestone were used. The salt crystallization tests were carried out in chambers at 23 ± 2 °C and 33 ± 5% relative humidity.

The trend of the results obtained for the Na_2_SO_4_ crystallization tests in soll, bajda, and safra were similar. Indeed, in all cases, the flow rate of the solution rising through inhibitor-treated slabs was greater than that rising in the non-treated ones; thus, the inhibitor was helping the crystallization to occur on the surface of the stone, and not within the pores. It is to be noted that the location of the salt crystals, or “drying front”, apart from being dependent on the pore size within the porous substrate (vide supra), relies on the balance between the rate of evaporation and the rate of solution replenishment at the site where the salts crystallize [2,62], meaning that if the latter is slower than the former, crystallization occurs within the stone as subflorescence, and can therefore cause damage.

A more pronounced effect of PC, relative to citrate, towards crystal growth inhibition was generally observed. This was attributed to the powerful crystal binding affinity generated by its strong negative charge/size ratio and a more favorable stereochemistry. Moreover, the data also showed a slight dependence on the concentration of the inhibitors, with 10 ppm yielding the optimal results. Besides, for the untreated stone, even after one salt cycle, faster and more pronounced deterioration was observed for the soll samples than for the franka ones, which was attributed, at least in part, to the difference in the pore size distribution. Particularly, porosimetry measurements (mercury intrusion porosimetry; MIP) indicated that soll exhibited the lower overall porosity (29.8%) when compared to bajda (30.4%) and safra (35.6%) but had a higher percentage of small pores, in the ranges 0.1–0.6 μm and 1–2 μm. For the franka, the maximum concentration of the distribution of pores lies in the range 2–4 μm [95]. This correlates well with the assumption that crystallization stresses are smaller in larger pores [33]; consequently, there is an elevated risk of greater forces in the smaller pores, together with a possible pore clogging effect (vide supra), generally resulting in more damage [33]. Furthermore, in soll, the formation of an external crust was observed both with and without the application of inhibitors, whereas in bajda and safra, damage consisted only of limited surface powdering. Interestingly, after the crystallization tests, when the debris brushed from the soll surface was weighed after separating it from salt, the loss of material registered a decrease of up to ~50% for inhibitor-treated samples vs. reference samples.

In a further contribution [79], we reported on the evaluation of the effectiveness of phosphocitrate (C_6_H_9_O_10_P; PC) in slowing the development of salt damage occurring in the Roman mosaic of Orpheus and the Beasts in Perugia, Italy (II century a. C.). To the best of our knowledge, this was the first in situ use of PC as a potential treatment for halting/mitigating the disruptive salt effects in natural porous media. This site, once a part of the public thermal baths, represents the myth of Orpheus enchanting animals with the sound of his lyre. Different areas of the mosaic, with a visually similar amount and distribution of salts, were treated with PC applied by cellulose poultice. Nearby, areas where a poultice using only distilled water was applied were used as a control. After a given period of time (three months) from the removal of the poultices, it was observed that the presence of PC had clearly delayed the new appearance of salts and modified the morphology of crystallizing salts from the encrusting type to whisker-like efflorescence, without damaging the mortar. Additionally, SEM analysis of the microsamples of porous mortar revealed that the morphologies of the salts precipitating inside the mortar itself were also influenced by the presence of the inhibitor. Specifically, it was observed that in the mortar from the control area, salt precipitated mainly as massive elongated large crystals, whereas in the presence of the inhibitor, rounded and significantly smaller crystals of gypsum (CaSO_4_·2H_2_O) and sodium sulfate (Na_2_SO_4_) were visible. In other words, a different crystal habit led to different tensile stress owing to the different surface contact between the crystals and the pore [31,33]. Importantly, besides all of these effects, an enhancement in the desalination efficiency due to PC was also observed. In fact, conductivity measurements clearly indicated that applying a poultice made by soaking a phosphocitrate solution led to a better extraction capacity than that based on pure water (~2x). This result suggests the possible use of this inhibitor for improving desalination treatments. Of course, many other important aspects, including thermal stability, active lifetime, re-treatability, and performance under complex salt mixtures, must be investigated before the true potential of PC can be established. Most recently, some of us patented [96] the formulation of an eco-compatible coating composition in the form of an aqueous suspension based on PC and zinc oxide micro- and/or nano-sticks, with combined inhibitory and antimicrobial activity towards salt deposit formation. The composition is particularly suitable to be used for the durability of porous materials, such as plasters, mortars, tuff, concrete, etc. It is noteworthy that among the solutions available in the market, no coating composition exhibiting salt formation inhibition and an antimicrobial effect at the same time exists.

#### 3.2.2. Phosphonates

In 2007, Lubelli and van Hees [97] reported a systematic investigation of the salt inhibition effects of both diethylenetriaminepentakis methylphosphonic acid (C_9_H_28_N_3_O_15_P_5_; DTPMP) and sodium-ferrocyanide (NaFe(CN)) when applied in 0.001 M concentrations to 10 wt% sodium sulfate (Na_2_SO_4_) and sodium chloride solution (NaCl) in three different materials, i.e., Granada limestone, a Czech sandstone, and a fired-clay brick. The results for the NaFe(CN) experiments will be discussed later in this review (see Section 3.3.).

On the basis of previous studies indicating that the maximum inhibition effect of the DTPMP was in slightly alkaline environments (vide supra) [51], the pH of the inhibitor solution was adjusted to 8. Interestingly, it was found that the effectiveness of DTPMP in enhancing salt solution transport strongly depends on the material to which it is applied. DTPMP promoted salt solution transport in sandstone, leading to a notable increase in the amount of salt transported to the surface; on the other hand, it delayed it in the fired-clay brick, leading to the precipitation of a greater amount of salt in depth, and had no effect in limestone. The authors speculated that the alkaline environment of the investigated limestone might have limited the effectiveness of this inhibitor. Besides, the phosphonates exhibited a strong tendency to adsorb onto a large variety of surfaces, e.g., calcite (CaCO_3_) (main component of the limestone), and aluminum and iron oxides (present in large amounts in the fired-clay bricks). It was on this basis that they explained the limited effect of DTPMP in limestone and brick. Finally, it was demonstrated that DTPMP was not able to prevent or delay the decay in any of the selected substrates, except for a slightly positive effect on Na_2_SO_4_ damage for the Granada limestone.

San Jeronimo monastery (Granada, Spain) [98] was selected as a case study for the on-site testing of a treatment using a DTPMP crystallization inhibitor. The monastery was chosen taking into account the extreme salt damage affecting the building stones, i.e., a biomicritic limestone, calcarenite, and wall paintings. An integrated approach combining multi-technique analysis, phenomenological observations, salt and moisture analysis, environmental monitoring, and simulation techniques was employed to study the salt damage problems affecting this building. Mostly, the salts were found to be magnesium sulfate in the form of either hexahydrite (MgSO_4_·6H_2_O) or epsomite (MgSO_4_·7H_2_O) depending on the climate conditions, together with minor amounts of gypsum (CaSO_4_·2H_2_O), nitrates, and chlorides.

Thus, focusing on the inhibitor, within seven months from the application of DTPMP in the test areas, it was demonstrated that the amount of efflorescence developed in the control area was greater than that in the treated area. This correlated well with the laboratory results indicating that DTPMP acts as crystallization inhibitor when it is free in solution, because it is able to establish hydrogen bonds with epsomite water molecules, and exhibits a high stereochemical affinity with Mg atoms in their dominant surfaces, as shown by molecular modeling; however, in the stone, it seems to promote the growth of subflorescence but without increasing damage to the support [99,100]. This was attributed to the adsorption of phosphonates on calcite surfaces. In fact, adsorbed phosphonate molecules can act as a template for magnesium sulphate crystallization, and consequently, crystallization of epsomite takes place at lower supersaturation within the stone pore network.

Nevertheless, the authors stated that it is still premature to draw conclusions on the potential positive/negative effects of the DTPMP inhibitor on MgSO_4_ crystallization damage based on a single case study.

Ruiz Agudo et al. [101] studied the template-assisted crystallization of sodium and magnesium sulfates (Na_2_SO_4_ and MgSO_4_) in the presence of Iceland Spar (calcite, CaCO_3_) single crystals and within limestone “directed” by diethylenetriamine-pentakis (methylphosphonic acid) (DTPMP). In order to better understand the phosphonate-directed crystallization of the two inorganic salts onto calcite, the authors combined macroscale (X-ray diffraction, XRD, and nuclear magnetic resonance, NMR) and microscale (atomic force microscopy, AFM, and environmental scanning electron microscopy, ESEM) techniques for the in situ monitoring of such crystallization. It was demonstrated that DTPMP, in the presence of MgSO_4_ in solution, strongly adsorbed onto the crystalline substrate but presented very weak interactive forces with MgSO_4_. On the other hand, in the presence of Na_2_SO_4_, an irreversible process involving the formation of a precipitate on the calcite surface, most probably crystalline Ca-phosphonate, could be observed. The authors explained this effect through the complexation effect that DTPMP has towards Mg^2+^ ions. This may prevent or inhibit the precipitation of Ca-phosphonate; moreover, Mg^2+^-phosphonate complexes are generally more stable and soluble than Ca^2+^ complexes. Atomic force microscopy (AFM) dynamic experiments confirmed this hypothesis and showed that the adsorption of DTPMP molecules halts the spreading of salt pits and prevents the formation of new ones, so that the DTPMP acts as a calcite dissolution inhibitor. The ESEM and XRD results combined with the morphology simulation showed that, in the presence of DTPMP, Na_2_SO_4_ crystallizes as mirabilite (Na_2_SO_4_·10H_2_O), with (001) faces aligned parallel to (101̅4) calcite planes. This could be due to the 2D-heterogeneous nucleation of mirabilite onto the substrate due to a thin layer of Ca-phosphonate on the {101̅4} calcite surfaces. Similar results were found for MgSO_4_, but in this case, the XRD pattern showed that apparently, the interaction between the additive and hydrated magnesium sulfates was not highly specific and must have taken place through the formation of hydrogen bridges between the functional groups of the additive adsorbed onto calcite and the structural water molecules in the magnesium sulfate crystals. Indeed, adsorbed DTPMP molecules were able to “direct” both hexahydrite (MgSO_4_·6H_2_O)- and epsomite (MgSO_4_·7H_2_O)-oriented crystallization. In the case of hexahydrite, (4̅11) planes showed a high density of water molecules. This was also valid for (010) epsomite planes. This makes these planes ideal for additive–salt crystal interaction.

The same authors also studied the effect of DTPMP within limestone pores. In this case, a strong reduction in the salt-induced damage and salt pore filling (by mercury intrusion porosimetry; MIP) was observed both for Na_2_SO_4_ and MgSO_4_ in the presence of DTPMP. The results showed that the crystallization of sulfates was promoted, thus taking place in a compact and oriented manner (as observed by in situ ESEM and XRD experiments), at a lower supersaturation (as determined by NMR measurements). The calculation of the critical supersaturation and the actual maximum crystallization pressure, which was possible through the determination (NMR) of the maximum Na concentration values, showed that a significant reduction in crystallization pressure (4.23 vs. 1.68 MPa) took place in the presence of DTPMP in the case of mirabilite crystallization. The tensile strength of the tested limestone was close to the usual value for this limestone, so the observed reduction of damage was explained. The damage reduction experienced by the limestone blocks subjected to MgSO_4_ macroscale crystallization in the presence of DTPMP was explained by a similar mechanism as that proposed for the case of Na_2_SO_4_. Unfortunately, the technical limitations of NMR prevented the evaluation of the Mg concentration at the onset of crystallization, so the authors could not calculate the critical supersaturation and associated crystallization pressure of magnesium sulfate salts formed in the pores of the limestone.

### 3.3. Alkali Ferrocyanides

In 2002, Selwitz and Doehne [102] described the effect of potassium ferrocyanide (K_4_Fe(CN)_6_·3H_2_O; KFe(CN)) on the capillary passage of dilute and concentrated solutions of sodium chloride (NaCl) and sodium sulfate (Na_2_SO_4_) (5 and 20 wt%) through specimens of limestone, i.e., Monks Park limestone and Texas Creme limestone, at 43% relative humidity (RH). In the absence of inhibitors, aqueous NaCl flow through Monks Park limestone (18.6% porosity) gave predominantly subflorescence (~90%), and slight edge erosion, whereas Na_2_SO_4_ mainly effloresced and severely damaged the stone samples. When the moderately high-porous Texas Creme limestone was used (21.3% porosity), essentially only efflorescence occurred in all cases, with nearly no stone damage. The addition of 0.1–1% potassium ferrocyanide to the NaCl experiments employing Monks Park limestone significantly changed the nature of the flow patterns and deterioration. At both the investigated salt concentrations, all the solution entered the stone at rates faster than trials without the additive. Most of the NaCl emerged as thin filament-like efflorescence. Importantly, all the samples, after brushing away the efflorescence, were found to be undamaged. When the KFe(CN) content was lowered at the 0.01% concentration, a result very similar to that without using the ferrocyanide was obtained. This indicated a critical concentration for this inhibitor between 100 and 1000 ppm.

Attempts to evaluate the effect of KFe(CN) on Texas limestone exposed to NaCl and Na_2_SO_4_ solutions failed, since the latter caused no apparent salt damage under all the investigated experimental conditions.

**Figure 7:**
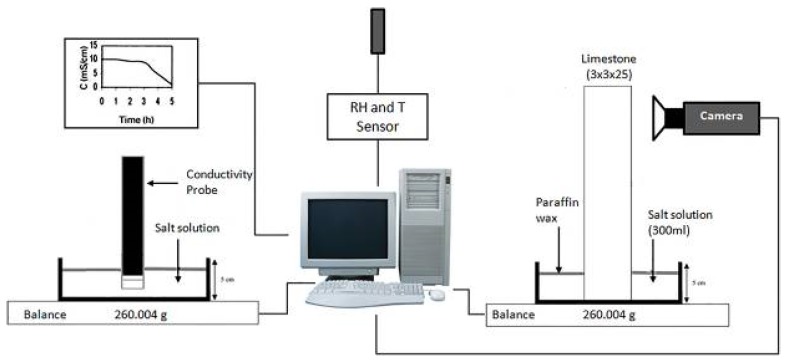
Experimental set-up for crystallization experiments in solution (left side) and within the porous stone (right side), as employed by Rodriguez-Navarro et al. [102].

Shortly thereafter, Rodriguez-Navarro and coworkers reported a series of systematic investigations to assess the efficiency of Na and K ferrocyanides (Na_4_Fe(CN)_6_·10H_2_O and K_4_Fe(CN)_6_·3H_2_O; Fe(CN)s) in minimizing sodium chloride (NaCl) crystallization damage in porous materials [103]. Specifically, the effect of Fe[(CN)_6_]^4−^ ions on the crystallization of NaCl in aqueous solution was studied, allowing the saline solution to percolate through and evaporate from a biomicritic limestone (calcarenite), typical of Granada (Spain) (Figure 7). Sodium and potassium ferrocyanide were added separately in concentrations ranging from 0.01% to 0.1% to the NaCl saturated solution. The crystallization tests were performed in a controlled environment (20 ± 1 °C, and 45 ± 5% relative humidity).

The authors first performed batch crystallization experiments. The addition of Fe(CN)s (from 0.01% to 0.1% *w*/*w*) did not change the NaCl solubility, nor did they modify the saline solution evaporation rate. However, significant increases up to ~7x for 0.1% Fe(CN)s were demonstrated for the critical supersaturation (i.e., maximum relative supersaturation reached before the onset of crystallization).

Conductivity measurements demonstrated a clear difference in the onset of NaCl crystallization in the absence and in the presence of additives; specifically, while in the former case, the NaCl crystallization onset corresponded to a significant drop in the conductivity, in the latter case, no relevant change was detected after the onset of NaCl crystallization, thereby suggesting that a significant concentration of additive was still available to prevent further NaCl nucleation, enabling a very high supersaturation. These results suggested that nucleation inhibition, which was also found to depend on the additive concentration, was the prevailing mechanism of additive–NaCl interaction. In addition, the evaporation rate of the solution, with additives moving by capillary rise through the stone specimens, was much higher than that of a pure NaCl solution. The authors attributed this to a reduction of the solution surface tension (vide supra).

Alternatively, a reduction in the solution–stone contact angle due to ferrocyanide adsorption onto the stone pore walls (CaCO_3_, calcite) could also have led to a faster capillary supply towards the evaporation front, thus promoting evaporation. The authors also stated that the rapid development of a dendritic efflorescence on the stone surface in the presence of ferrocyanide contributed to the increase of the evaporation rate by creating a porous medium with a high surface area.

A massive formation of efflorescence was explained by the demonstrated inhibiting role of ferrocyanide on NaCl nucleation, which prevented this salt from crystallizing within the pores of the stone. As to the stone damage, some NaCl crystals in the control run were found to crystallize as subflorescence, inducing granular disintegration at the stone upper edges and corners (i.e., areas of faster evaporation). However, in the presence of ferrocyanide, negligible subflorescence occurred, with almost all crystallization taking place on the stone surface; no damage was therefore detected in this latter case, although the higher supersaturation reached in the presence of Fe(CN)s would, theoretically, result in higher crystallization pressure. It is worth pointing out that SEM (scanning electron microscopy) and XRD (X-ray diffraction) analysis demonstrated that both sodium and potassium ferrocyanides induced similar and significant NaCl crystal habit modifications in stone efflorescence, from the most common cubic halite to dendritic growth. The additives were therefore demonstrated to play a role in delaying the incorporation of the solute ions into any face of a growing crystal, thereby increasing the solution supersaturation, as well as changing the relative growth rate of the different faces following their adsorption.

Lubelli and van Hees [97], in 2007, demonstrated the inhibition effects of sodium-ferrocyanide (Na_4_Fe(CN)_6_·10H_2_O; NaFe(CN)) when added in a 0.001 M concentration to a sodium chloride (NaCl) solution (10 wt%) in three different materials, i.e., Granada limestone, a Czech sandstone, and a fired-clay brick (vide supra). Interestingly, it was found that the inhibitor enhanced NaCl solution transport in both the Spanish limestone and in the fired-clay brick, whereas it had no significant effect in the sandstone. Notably, the ferrocyanide was very effective in changing the morphology of the halite; in fact, in the presence of NaCl only, a dense formation of cubic halite crystals was found on the surface, while by adding the inhibitor, efflorescences turned into a branched shape, thus having a much larger evaporation surface. Sodium ferrocyanide behaved differently in Czech sandstone, and the authors attributed this to the presence, in the latter, of large pores in a very limited diameter range (20–30 µm). More specifically, they hypothesized that the continuous liquid network needed for a salt solution transport would be broken sooner than in materials having a wider distribution of pore sizes, in which a network of pores of a smaller radius could guarantee the liquid’s transport for a longer time. As a result, the salts would precipitate in the pores. The effect of the inhibitor would in this case be limited. Following this, the authors assessed the salt distribution by measuring the hygroscopic moisture content to confirm the effectiveness of the inhibitor in enhancing salt solution transport. They found that in the Granada limestone as well as in fired-clay brick, the presence of sodium ferrocyanide enhances the transport of salts to the surface and therefore their crystallization as efflorescence instead of cryptoflorescence. In the Czech sandstone, the ferrocyanide did not show any significant effect. The morphology of NaCl precipitating inside the stone was also influenced by the presence of the inhibitor, i.e., in the absence of inhibitor, halite precipitated mainly as a layer on the pore wall, whereas in the presence of the inhibitor, an agglomeration of small crystals was visible, filling the pore spaces.

In conclusion, however, the effectiveness of sodium ferrocyanide in preventing NaCl crystallization damage could not be proven by the above experiments. In fact, the crystallization tests did not cause any decay, both in substrates treated with the inhibitor as well as in untreated substrates.

In a further interesting contribution, Lubelli and coworkers [104], in 2010, presented a pilot study in which sodium ferrocyanide (Na_4_Fe(CN)_6_·10H_2_O) was mixed in a newly prepared lime-cement mortar. The salt resistance of the hardened mortar was tested by means of crystallization experiments. It is to be noted that the inhibitor was mixed in the water used to prepare the mortar (0%, 0.05%, 0.5%, and 1% relative to water). The results of the crystallization tests showed that the inhibitor significantly improved the salt resistance of mortar subsequently contaminated with sodium chloride (NaCl). Additional observations revealed that the amount of efflorescence increased with the inhibitor content. In addition, an effect of the inhibitor on the NaCl crystal morphology was also observed. Indeed, the salt was found to form feather-like dendritic crystals that poorly adhered to the surface. These were clearly different from the strongly adhering more massive efflorescence, observed on reference specimens in which the crystal featured a conventional cubic habit; the amount of material loss in a subsequent crystallization test under laboratory conditions was up to 100 times less than in a mortar without inhibitor. The reduction of material loss was accompanied by different macroscopic damage patterns: Mortar without inhibitor showed sanding, bulging, and scaling, whereas the mortar with inhibitor only showed sanding. An inhibitor content of 0.5% (of the weight of the water used to prepare the mortar) was enough to significantly reduce the damage. The use of higher amounts did not give any further improvement.

The reduction in the damage was attributed to the appearance of efflorescence in the presence of inhibitor, instead of in-pore crystallization. SEM (scanning electron microscopy) observations carried out on the surface of the samples demonstrated that the inhibitor modified the crystal habit of the salt crystals as well as inhibiting the development of specific crystal faces. Indeed, the NaCl crystals at the surface of the specimen without inhibitor formed a compact mass with regularly shaped large cubic crystals. The presence of the inhibitor gave the crystal a spongy appearance, with less surface to adhere to the pore walls. The authors argued that the development of a spongy habit showed that the inhibitor became effective when the crystallization was ongoing, and not at the onset of crystallization. In addition, the presence of the crystallization inhibitor seemed to favor the preferential development of specific crystal faces, resulting in elongated prismatic crystals.

Rivas and co-workers [105], also in 2010, evaluated the influence of sodium and potassium ferrocyanides (Na_4_Fe(CN)_6_·10H_2_O and K_4_Fe(CN)_6_·3H_2_O; Fe(CN)s) on sodium chloride (NaCl) crystallization in two granitic rocks used in the past or the construction of heritage buildings in southern Galicia (northwest Spain), i.e., Monçao and Rodas granites. It is to be noted that the rocks selected for this study were rather different in terms of the porous structure. Indeed, the fraction of the pores accessible to water was much less in Monçao than in Rodas granite (~0.97 vs. ~5.90). Consequently, Monçao granite exhibited a lower porosity by Hg injection (mercury intrusion porosimetry; MIP) than that for the Rodas one (~4.34 vs. ~6.94). Additionally, the pore size distributions revealed that in Monçao granite, the micropores (0.1–0.01 µm) accounted for only 10% of the total porosity, whereas in the Rodas granite, they reached 63%. On these bases, the authors claimed that the marked difference between the MIP porosity and the water-accessible porosity in Monçao rock indicated an inefficient water transport across its porous network. Subsequently, the evaporation rate of the saline solutions migrating through the rocks was determined for pure NaCl solutions (26.4 and 18 wt%), for sea water (to simulate more complex solutions), and for both aqueous solutions with inhibitors at 0.1% and 0.01%. It was demonstrated that the two rocks showed very different behavior. In the Rodas granite, the evaporation of the pure solutions of NaCl was accelerated in the presence of Fe(CN)s and took place in proportion to the concentration of ferrocyanide in the water. It was also observed that the solutions that evaporate most rapidly were those most concentrated in NaCl (26.4 wt%), and no differences between the two ferrocyanides could be observed. Similarly, in the case of Monçao granite, the Fe(CN)s increased the rate of evaporation of the pure solutions of NaCl, though a clear effect was observed only in the presence of a 0.1% concentration of ferrocyanide. Again, no differences between the two ferrocyanides were found. On the other hand, the solutions that evaporated most rapidly in this rock were those dilute in NaCl (18 wt%). In the case of sea water, the different behavior between the Rodas and Monçao rocks was confirmed. Particularly, in the Rodas granite, the inhibitors accelerated the evaporation of the sea water, the most efficient inhibitor being sodium ferrocyanide. Furthermore, the evaporation rate of the sea water was lower than that for the pure solutions of NaCl. On the other hand, for the Monçao granite, a very different behavior between one test piece and another of the same series was found, thereby making it difficult to conclude that the inhibitors did in fact accelerate the evaporation of sea water. In general, the ferrocyanides did not appear to significantly alter the evaporation rate. Efflorescence formed during evaporation of the solutions with and without inhibitors were analyzed by, e.g., scanning electron microscopy (SEM) and X-ray diffraction (XRD).

In both granites, the deposits formed (halite) in the absence of inhibitors were generally massive and of a very hard consistency; in the presence of the inhibitors, efflorescence consisted of small crystals of dendritic habit. However, in Rodas granite, the ratios between efflorescence and subflorescence obtained when solutions of pure NaCl without modifier were evaporated were lower than those obtained in the presence of ferrocyanides, thereby indicating a positive effect of the inhibitors in promoting NaCl crystallization on the surface rather than below it. In Monçao granite, the results were completely different, i.e., the ratio between efflorescence and subflorescence was lower in the presence of the modifiers than in the pure NaCl solution. Finally, different behavior between the two rocks was also observed with respect to the evaporation of the pure solutions of NaCl without modifiers: In Rodas granite, the rate of evaporation and the efflorescence/subflorescence ratios were greater in the case of the concentrated NaCl solution; however, the opposite was found in the Monçao stone, where the rate of evaporation and the ratio between efflorescence and subflorescence were greater for the 18% NaCl solutions. Interestingly, with sea water, very different results were also obtained for the two rocks: In the Rodas, the efflorescence/subflorescence ratios were greater in the presence of the inhibitors, indicating that even in mixed solutions, ferrocyanides promoted the migration of sodium chloride towards the surface. However, it was found that this efficiency was greater when the ferrocyanides were in a lower concentration, which is contrary to what occurs with pure solutions. In the Monçao granite, it was again found that the above ratios were lower in the presence of the modifiers, indicating that, also for this type of solution, the inhibitors not only did not act but also even promoted the opposite effect to that desired, that is, crystallization below the surface. It is important to note here that no other salts that might have crystallized from sea water (e.g., magnesium sulfate) were found in the efflorescence, thereby indicating that the inhibitors are able to alter not only the crystallization kinetics of NaCl but also that of other salts. The authors also interestingly found that the addition of ferrocyanides to distilled water appeared to increase the effectiveness of desalination by immersion of the Rodas granite, relative to the use of distilled water only, particularly for the first 1.0–1.5 cm of depth (50–70% vs. 34–52% of chloride extraction, respectively), with sodium ferrocyanide giving the best performance.

Additionally, Gupta and co-workers [106], in 2012, focused on the influence of ferrocyanide ions on the crystallization behavior of sodium chloride (NaCl), moisture and ion transport, and salt damage in porous materials. The authors investigated the effect of potassium ferrocyanide (K_4_Fe(CN)_6_·3H_2_O; KFe(CN)) on the concentration levels reached by NaCl solutions within porous media during drying, and the associated water and ion transport. To asses this, using a specially designed nuclear magnetic resonance set-up [106] (see Section 4.1.2.), two types of drying experiments were performed: The first series was on droplets of salt solution with and without inhibitor; and the second on porous materials (fired-clay brick and Granada limestone) contaminated with salt solution with and without inhibitor. It is noteworthy that the experimental set-up enabled the nondestructive measurement of both hydrogen and sodium ions simultaneously during the drying experiments. The theoretical background of these measurements is the so-called advection diffusion equation (Equation (3)):

(3) $$\frac{\partial C\theta }{\partial t}=\frac{\partial }{\partial x}\left[\theta \left(D\frac{\partial C}{\partial x}-CU\right)\right],$$

where *C* is the concentration given in molL^−1^, *θ* (m^3^m^−3^) is the volumetric moisture content, *D* (m^2^s^−1^) is the diffusion coefficient of salt ions in porous matrix, *t* (s) is the time, *x* (m) is the position, and *U* (ms^−1^) is the velocity of the fluid.

**Figure 8:**
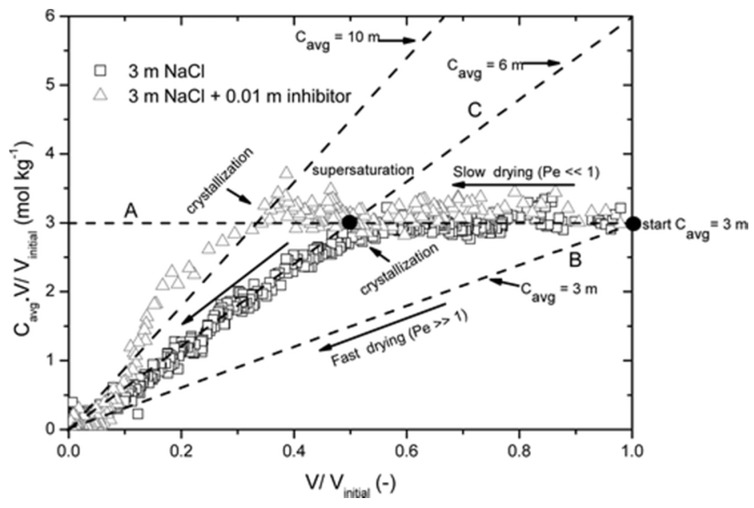
Advection−diffusion analysis diagram for the droplet drying experiment: The total amount of dissolved sodium in the droplet is plotted as a function of the volume of the droplet (*V*). Both the axes are normalized with respect to the initial volume of the droplet (*V_initial_*). The division of both the axes gives the average concentration (*C_avg_*) of Na in the NaCl solution droplet shown by solid lines in the figure. The results for 3 m NaCl salt solution droplet with (Δ) and without inhibitor (□) are shown, by Gupta et al. [106]. Reproduced with permission from [106]. Copyright (2012) American Chemical Society.

This equation shows the competition between advection, which transports ions to the top of the sample and thereby causes accumulation, and diffusion, which levels off accumulations. This competition is given by the Peclet number (*Pe*), defined as in Equation (4):

(4) $$Pe=\frac{\left|U\right|L}{D},$$

with *L* (m), the length of the sample, and *D*, the diffusion coefficient for dissolved NaCl (1.3 × 10^−9^ m^2^s^−1^). Measuring the moisture profiles, the authors calculated the solution velocity. For *Pe* << 1, diffusion dominates, and the ion-profiles will be uniform, whereas for *Pe* >> 1, advection dominates, and ions will be accumulated at the drying surface. The amount directly measured by NMR (nuclear magnetic resonance) was plotted in the so-called efflorescence pathway diagram (EPD) [107] (Figure 8).

Briefly, in this diagram, it is possible to identify two limiting situations. First, in the case of very slow drying (i.e., *Pe* << 1), the ion profiles are uniform; at a given point, the average NaCl concentration slowly increases (line A) up to the saturation concentration (6.1 m; m = molal). From this point, any further drying will lead to crystallization (line B) and the concentration is constant at 6.1 m. When very fast drying occurs (i.e., *Pe* >> 1), ions are directly transported by advection with the moisture to the top of the sample, and a 6.1 m peak will build up with a negligible width; that is, the average concentration is almost not affected at all. If the crystallization rate is fast enough, i.e., if at the top of the sample there are sufficient nucleation sites, the average NaCl concentration in the solution in the sample itself will be constant and close to the initial concentration (line C). From any point within the lines A–C, the moisture removal only will lead to an NaCl concentration increase.

Based on the droplet drying experiments, the authors concluded that the inhibitor enhanced the rate of the drying process, thereby leading to an increase in efflorescence. Moreover, they observed a higher supersaturation and change in the crystal patterns. In the brick drying experiments, the inhibitor also determined a change in the NaCl crystal morphology.

This led to a change in the drying conditions at the material–air interface. Indeed, we have already highlighted here that dendrite-like crystals provide a much higher surface area for evaporation, which ultimately make the advection a governing phenomenon during drying.

In a subsequent paper, the same group [108] reported an analogous study focusing on fired-clay bricks as porous media. The relative humidity was varied inside the NMR chamber at 0%, 55%, and 70%. At the end of each drying experiment, the efflorescence from the sample surface was collected and weighed. The authors demonstrated that the evaporation rate in the salt-loaded fired-clay brick was greater for the higher humidity; this was explained by considering that at low humidity, advection is the dominant process in the initial stage of the drying process, and therefore, under these conditions, ions crystallize fast and block the surface. The addition of ferrocyanide inhibitor was found to be useful at low humidity conditions, since salt (NaCl) crystallized as nondestructive efflorescences. At a high humidity, salt ions crystallized slowly outside the material as efflorescence, thereby preventing pore clogging. As a consequence, the addition of inhibitor did not show any significant effect on the material drying behavior, amount of efflorescence formed, moisture, or ion transport on the salt concentration levels inside the brick.

More recently, in 2015, Gupta et al. [109] extended their investigations to salt mixtures, such as sodium-potassium chloride (NaCl–KCl) and sodium-lithium chloride (NaCl–LiCl). In these cases, the authors carried out drying experiments only on solution droplets, with and without ferrocyanide as the crystallization modifier at different concentrations. This study demonstrated that the increase in supersaturation by inhibitor addition was smaller for salt mixtures compared to single salt (NaCl, vide supra). On the other hand, similarly to the single salt case, the morphology of NaCl crystals in salt mixtures changed dramatically, going from bigger and strongly adhered cubic crystals to smaller and loosely attached dendritic crystals that promoted efflorescence and led to negligible structural damage of the material.

### 3.4. Surfactants

Rodriguez-Navarro and coworkers [110] studied the effect of two types of ionic surfactants, i.e., anionic sodium dodecyl sulfate (C_12_H_25_NaO_4_S; SDS) and cationic cetyldimethylbenzylammonium chloride (CH_3_(CH_2_)_15_N(Cl)(CH_3_)_2_CH_2_C_6_H_5_; CDBAC), on the process of sodium sulfate (Na_2_SO_4_) crystallization in a porous calcareous stone. A greater evaporation rate of sodium sulfate solution with SDS was observed compared both to the blank sample and, particularly, the solution with CDBAC, thus resulting in a faster transport towards the evaporation front, located a few millimeters below the limestone surface. Mercury intrusion porosimetry (MIP) analysis revealed that both anionic and cationic surfactants induced salt crystallization within the stone pore network, the porosity reduction being more significant in the case of the cationic surfactant. No porosity changes were detected in the blank following salt crystallization, meaning that salts concentrated in the surface scales formed and fell off with the stone debris (vide infra), without clogging the pores of the rest of the stone samples. In situ ESEM (environmental scanning electron microscopy) observations of Na_2_SO_4_ crystallization in the limestone showed that in the blank set-up, crystallization of mirabilite takes place as aggregates of hollow-faced or prismatic crystals, corresponding to crystallization at high supersaturation (i.e., crystal shapes are far from the equilibrium shape, vide supra).

On the other hand, in the presence of SDS, mirabilite (Na_2_SO_4_·10H_2_O) growth occurred as rhombohedral or prismatic or isolated hopper crystals, also indicative of crystallization at high supersaturation ratios (i.e., high crystallization pressures), whereas in the presence of CDBAC, Na_2_SO_4_ decahydrate crystallized as euhedral, bulky, and rhombohedral mirabilite crystals formed at low supersaturation ratios, i.e., featuring lower crystallization pressures, filling the pores of the stone. It is also to be noted that in the case of dehydration/hydration cycles, significant damage in the sample with salt and cationic surfactant was observed.

Consistently, crystallization of mirabilite in stone specimens resulted in scale formation and significant stone loss in the blank (30 wt% loss) as well as in the slab subjected to crystallization in the presence of SDS (32 wt% loss) whereas somewhat reduced damage occurred in the presence of CDBAC (28 wt% loss). The authors claimed that the cationic surfactant, being concentrated in the bulk of the saline solution, due to its demonstrated low adsorption onto calcite vs. Na^+^, formed a large amount of micelles, which might have induced the nucleation and growth of sodium sulfate at low supersaturation ratios by solubilizing the solutes and promoting their transport towards mirabilite nuclei growth sites. It is to be noted that the even distribution of mirabilite crystals throughout the stone pore system might eventually result in significant damage if dehydrated mirabilite re-hydrates. On the other hand, the anionic surfactant was less concentrated in the saline solution because of its preferential adsorption onto calcite (CaCO_3_), in addition to being precipitated as sparingly soluble calcium dodecylsulfate (Ca(DS)_2_). Consequently, a higher supersaturation ratio was achieved.

Importantly, this study demonstrated that an impregnation with the above ionic surfactants was not an effective method for stone desalination when hydrated salts are present, since side effects can be serious (i.e., cationic surfactant).

Table 1 presents, in summary form, the information presented so far in the above sections.

**Table 1:** Summary of the most representative inhibitor families used to date, and their related effects.

	Inhibitor Type (Concentration)		Salt Type (Concentration)	Effects	Ref.
Biomass-derived	CA[a] 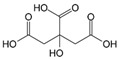	10^−6^ M to 10^−4^ M	Na_2_SO_4_ (0.7 M or 0.35 M)	enhanced solution flow rate through tuff with inhibitory activity slightly higher than that of phosphorylated counterpart (PC); promotion of *efflorescence* growth as opposed to *subflorescence*; no effect in Sicilian limestone; enhanced solution flow rate through Globigerina limestone although with a lower inhibition than the phosphorylated counterpart (PC).	[49,75,77,78]
			NaCl (1.54 M)	enhanced solution flow rate through tuff with slightly lower inhibition capacity respect to the phosphorylated counterpart (PC); no effect through brick and Noto/Palazzolo limestone.	[76,77]
			NaCl+Na_2_SO_4_ (0.68+0.28 M; 1.03+0.14 M; 0.34+0.42 M)	enhanced solution flow rate through tuff; no effect on brick.	[76]
	PC[b] 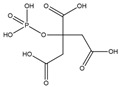	10^−6^ M to 10^−4^ M	Na_2_SO_4_ (0.7 M or 0.35 M)	enhanced solution flow rate through brick, tuff, Noto/Palazzolo limestone, Globigerina limestone; crystallization mainly on the materials surface as thin filament-like *efflorescence*; in *soll* f(Globigerina limestone) formation of an external crust and decrease of the material loss; in *bajda* and *safra* (Globigerine limestone) damage only as some surface powdering.	[49,75,77] [78,80]
			NaCl (1.54 M)	enhanced flow rate of the solution through tuff; no effect on brick.	[76,77]
			NaCl+Na_2_SO_4_ (0.68+0.28 M; 1.03+0.14 M; 0.34+0.42 M)	enhanced flow rate of the solution through tuff; no effect on brick.	[76]
Phosphonates	DTPMP[c] 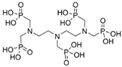	10^−3^ M	Na_2_SO_4_ (1 wt%) *	in Czech sandstone the drying and the amount of salt on the surface notably increased; in brick, inhibited salt solution transport, leading to a greater amount of salt in depth.	[97]
		10^−4^ M to 10^−2^ M	Na_2_SO_4_ (3.1 M)	2D-heterogeneous nucleation of *mirabilite* on the substrate due to a thin layer of a Ca-phosphonate on the {101̅4} *calcite* surfaces; crystallization promoted at lower supersaturation leading to a lower crystallization pressure.	[101]
			MgSO_4_ (2.1 M)	*hexahydrite* (4̅11) planes and (010) *epsomite* planes-oriented crystallization on {101̅4} *calcite* surfaces.	
Alkali ferrocyanides		10^−3^ M To 10^−2^ M	Na_2_SO_4_ (5 wt%; 20 wt%)	*faster* rates of salt solution movements; extensive *efflorescence* in large crystals.	[102]
	KFe(CN)[d] 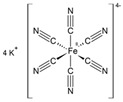		NaCl (5 wt%; 20 wt%)	faster rates of salt solution movement; formation of thin filament-like *efflorescence*.	[102]
		10^−4^ M to 10^−3^ M	NaCl (3 M); sea water	enhanced flow rate of NaCl solution in Rodas and Moncao granite leading to *efflorescences* with globular bunches of small dendritic crystals; enhanced flow rate of sea water solution only on Rodas granite; no effect on Moncao granite. Little or no change in NaCl crystals. However, differences occur with respect to the mineral phases formed: in the absence of the modifiers; MgSO_4_∙6H_2_O detected, but absent in the deposits produced in the presence of modifiers.	[105]
		10^−4^ M to 10^−2^ M	NaCl+KCl (2+2 M; 3+1 M); NaCl+LiCl (3+1 M; 2+2M; 1+3 M)	higher NaCl supersaturation that increases with increasing inhibitor concentration; for salt mixtures lower supersaturation compared to single salts; for both single salts and salt mixtures, crystal morphology changes from bigger and strongly adhered cubic crystals to smaller and loosely attached dendritic crystals.	[109]
		10^−3^ M To 10^−2^ M	NaCl (3 M)	faster drying rates with domination of advection phenomena that cause salt crystallization near the surface; dendritic crystal morphology increases the effective surface area for evaporation; at low humidity, increased nondestructive dendritic *efflorescences*; at high humidity amount of *efflorescence* similar with and without inhibitor.	[106] [108]
		10^−4^ M To 10^−3^ M		much higher evaporation rate of the solution; formation of a porous *efflorescence* with skeletal crystals with {110} faces and dendrities growing along <111> direction.	[103]
	NaFe(CN)[e] 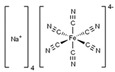		NaCl (3 M); sea water	the same effect seen for KFe(CN).	[105]
		10^−3^ M	NaCl (2 wt%) *	enhanced drying in Spanish limestone and brick; *efflorescence* assumes a branched shape.	[97]
		0% To 1%^†^	NaCl (3 M; 1.5 M)	greater amount of *efflorescences* with reduced material loss; small, elongated feather-like dendritic crystals with poor adhesion to the surface.	[104]
Surfactants	SDS[f] 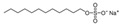	10^−3^ M	Na_2_SO_4_ (1.37 M)	higher evaporation rates of salt solutions; *mirabilite* growth as rhombohedral or prismatic or isolated hopper crystals; scale formation and significant stone loss.	[110]
	CDBAC[g] 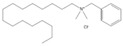			reduction in evaporation rates of salt solutions; *mirabilite* crystallize as euhedral, bulky, rhombohedral crystals filling the pores of the stone; reduced damage in stone	

[a] citric acid (C_6_H_8_O_7_); [b] phosphocitrate (C_6_H_9_O_10_P); [c] diethylenetriaminepentakismethylphosphonic acid (C_9_H_28_N_3_O_15_P_5_); [d] potassium ferrocyanide (K_4_Fe(CN_6_)·3H_2_O); [e] sodium ferrocyanide (Na_4_Fe(CN)_6_·10H_2_O); [f] anionic sodium dodecyl sulfate (C_12_H_25_NaO_4_S); [g] cationic cetyldimethylbenzylammonium chloride (CH_3_(CH_2_)_15_N(Cl)(CH_3_)_2_CH_2_C_6_H_5_); * weight of the salt/weight of the specimen; †relative to water used.

## 4. Experimental and Instrumental Techniques, Modelling

In the last two decades, capillary-rise and salt crystallization tests have been widely applied to evaluate the resistance of stones to the damaging action of salt crystallization. Other methods of investigation, such as pore-clogging and warping tests, differential scanning calorimetry (DSC), and dynamic mechanical analysis (DMA) [53], have been introduced over time to provide quantitative information on values of supersaturation for nucleation, kinetics of in-pore crystallization, pore clogging, crystallization pressure, and resulting material deformation [37].

Besides, several other analytical techniques have been used to understand the nature of the salts’ phase changes in solution or in the porous materials, e.g., environmental scanning electron microscopy and scanning electron microscopy (ESEM and SEM) [3,46,61,111], X-ray diffraction (XRD) [112] and environmental X-ray diffraction (RH-XRD) [113,114], electronic speckle pattern interferometry (ESPI) [114], nuclear magnetic resonance (NMR [52,115,116,117], and magnetic resonance imaging MRI [46,118]), and, more recently, synchrotron measurements [119,120,121] and atomic force microscopy (AFM) [42].

Salt weathering is a highly dynamic process, which is determined by the “coupling” of in-pore crystallization and dissolution, with the transport of heat, moisture, and salts through the pores under changing environmental conditions. Bearing this in mind, the development of numerical models has become ever more useful to compare the behavior of different salts, porous materials, or the effects of diverse climatic conditions. Currently, there are several models based on physical principles or empirical formulas [81,122,123,124,125]. For instance, Espinosa et al. [126,127,128] and Nicolai et al. [123] developed coupled three-dimensional numerical models for heat-water-salt transport and salt crystallization in building materials by using the control volume method (CVM). Koniorczyk and Gawin [129,130], Koniorczyk and Wojciechowski [131], and Koniorczyk [124,132] solved such a coupled problem by employing the finite element method (FEM) simulation. The three codes were founded on the study of Steiger (vide supra) as regards the description of the salt properties, and described salt crystallization on the basis of the kinetics of phase changes. Furthermore, Nguyen et al. [133] further modeled isothermal one-dimensional (1D) transport of moisture and salt, and salt crystallization; Paz-García et al. [134] described electrokinetic transport processes using FEM to model the desalination of porous building materials. Poupeleer [135] developed a 1-D CVM model based on poromechanics to calculate the deformation of building materials due to drying-induced salt crystallization; the model, however, did not enable prediction of the material cracking. Espinosa-Marzal and Scherer [136] used a poroelastic approach to describe 1-D deformations due to drying-induced crystallization at high temperatures. Here, also, the modelling of fractures was not possible. Finally, Derluyn et al. [125] gathered together the available approaches within a single unifying framework, and introduced a fully coupled model for heat, water, and salt ion transport, salt crystallization, material deformation, and damage caused by sodium chloride crystallization in a porous limestone.

This section will therefore focus on the experimental methods and instrumental techniques that have been used to gain insights into the mode of action of the inhibitors. We have split the discussion into two parts, i.e., macroscale and microscale determinations, summarized also in the table below (Table 2).

### 4.1. Macroscale Experiments

#### 4.1.1. Capillary-Rise Experiments

Currently, capillary-rise experiments are the most frequently used test to determine the efficiency of inhibitors. These experiments, based on the model of salt crystallization in a rigid porous substrate, proposed by Lewin [2], and then revised by Rodriguez-Navarro and Doehne [3], simulates the penetration of salts dissolved in groundwater into stone or masonry in situ, while evaporation takes place on the surface. Sodium sulfate (Na_2_SO_4_) is most frequently the reference salt used in the capillary-rise experiments, though sodium chloride (NaCl) is also often used, e.g., in Rodriguez-Navarro and Doehne [3] and Espinosa-Marzal et al. [53] (see Table 2); magnesium sulfate (MgSO_4_) has been occasionally investigated (Ruiz-Agudo et al.) [100]. Although this type of experiment does not allow for a quantitative analysis of the damage, it offers the possibility of comparing, at least qualitatively, the action of different salts, substrates, and even of eventual different preliminary treatments. Moreover, it is an ideal experiment for numerical simulation for salt crystallization coupled with salt transport in porous stone (Espinosa-Marzal and Scherer [37]), since it allows analysis of the influence of factors affecting the crystallization and damage patterns. This test, in a controlled environment, enables measurements of the evaporation rate, the crystallization pattern (XRD and SEM-ESEM coupled with energy dispersive X-ray microanalysis and/or cathodoluminescence analysis), and the distribution of the salts (MIP and HMC content) with as well as without the presence of crystallization modifiers in the porous medium. The changes in appearance and salt morphologies can be (and have been) documented by means of a time-lapse video (TLV) system and the extent of damage can be evaluated by weighing the material that falls off of the stone surface (after salt removal by immersion in distilled water), so that the total weight loss can be expressed as a percentage of the original weight of the stone block. Many authors, once the capillary-rise test is completed, usually determine, as a qualitative parameter of the efficiency of the additive, the efflorescence/subflorescence ratio, expressed as the ratio between the weight of the efflorescence formed and the weight of the sample.

Lubelli et al. [97] reported a very interesting variation of the capillary-rise experiment, aiming at reproducing as closely as possible the situation that may occur in practice during the application of a crystallization inhibitor. Thus, different from previously published experiments (e.g., Rodriguez-Navarro [103]), in which the modifiers are added to the saline solutions used for the tests, the inhibitor was applied by spraying it onto the surface of stone samples already contaminated by salts. The authors carried out these tests through wet-dry cycles. Based on the same rationale, another variation was reported in Marrocchi et al. [49,75,76,77,78] and Lubelli et al. [104], where the inhibitor was applied to the stone samples before their contamination with salt.

#### 4.1.2. Crystal Growth Experiments

Many authors have also carried out crystallization tests in solution, i.e., without the use of porous media. This was done to enable a better understanding of the inhibition mechanisms of the diverse crystallization modifiers, along with the influence of the diverse porous media on such a phenomenon. Typically, in these experiments, the salt solutions, with and without additive, were allowed to evaporate in open containers in an environmentally controlled atmosphere. The evaporation rate was determined by weight loss measurements. Conductivity measurements enabled determination of the induction time and critical relative supersaturation.

The induction time is defined as the interval between the start of the experiment and the onset of crystallization. The critical relative supersaturation, *σ*, is defined as the supersaturation reached at the onset of crystallization and is calculated as *σ = 100(C − C*_0_)/*C*_0_, where *C* and *C*_0_ are the actual and the saturation concentrations [103]. To compare different additives, Ruiz-Agudo et al. [51] normalized the critical supersaturation data with respect to the critical supersaturation of the control by using the percentage of growth inhibition (*GI*), calculated from the following equation:

(5) $$GI\left(\%\right)=\frac{\sigma_{additive}-\sigma_{blank}}{\sigma_{blank}}\times 100,$$

where *σ_additive_* and *σ_blank_* represent the critical relative supersaturations in the presence and in the absence of the additive, respectively, measured by conductivity. Moreover, changes in the viscosities, contact angles, and surface tension of the salt solution comprising the inhibitors were measured in order to establish if the additive had modified the salt solution properties. Indeed, a reduction of the solution surface tension could lead to a reduction of the capillary pressure *P* in a pore of radius *r* (Laplace’s equation), inducing a faster evaporation. Conversely, a reduction in solution–stone contact angle due to additive adsorption onto the stone pore walls could lead to a faster capillary supply toward the evaporation front, thus promoting evaporation. The correlation between the rate of capillary rise, the viscosity, the contact angle, and the surface tension is described by the Washburn’s equation [139]. At the end of the crystal growth experiments, the precipitates were collected and observed by means of ESEM and XRD to establish the crystal morphology and the crystallization form. On the other hand, FTIR, AAS, EDX, and CTL spectroscopy analysis were used to qualitatively assess whether irreversible adsorption of the additive onto the growing crystals occurred.

**Figure 9:**
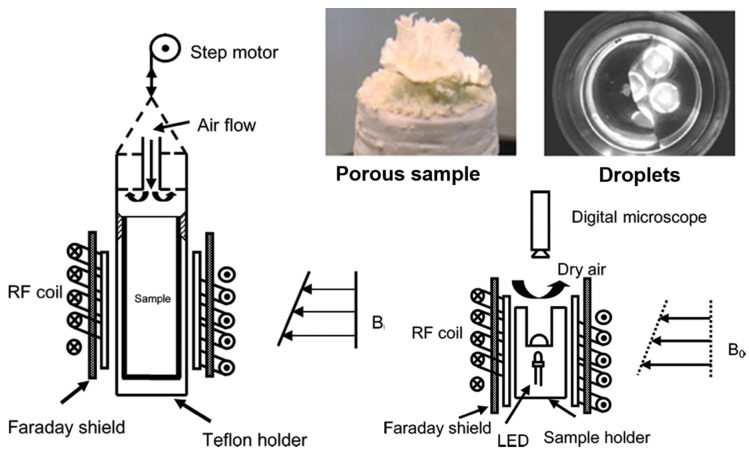
A schematic diagram of the NMR setup used for droplet drying experiments (right) and for the brick drying experiments (left) [106]. Reproduced with permission from [106]. Copyright (2012) American Chemical Society.

More recently, Gupta et al. [106] and Ruiz-Agudo et al. [101], using a specially designed nuclear magnetic resonance set-up (NMR) [106], were able to non-destructively measure the moisture distribution and salt concentration during saline solution evaporation and crystallization both in solution droplets and within the material’s pores during drying experiments. The set-up adopted by the authors is shown in Figure 9.

Focusing on the solution droplet, a cylindrical polymethyl-methacrylate (PMMA) sample holder was used and time lapse microscopy of the crystallization was performed using a digital microscope with four light emitting diodes (LEDs) placed below the substrate as a lighting source for imaging within the NMR set-up. The photomicrographs together with the NMR measurements allowed visual detection of the drying of the droplet and, at the same time, the gathering of information on the salt concentration of the droplets. In the case of porous specimens, they were first saturated by capillary rise with salt solution both with and without inhibitor. These samples were then sealed on all sides, except for the top surface, and placed in the NMR sample chamber, where the sample position was moved vertically to allow measurement of the moisture and ion content throughout the sample length.

Because of experimental constraints, only the Na and H concentration in solution could be measured. Therefore, the evolution of the salt concentration within the pores in the presence/absence of additives could only be studied for sodium sulfate (Na_2_SO_4_) [101] and sodium chloride (NaCl) [106] crystallization. Maximum Na concentration values determined by NMR enabled the calculation of the critical supersaturation and the actual maximum crystallization pressure that can be exerted by salt crystallizing within the porous stones using the equation proposed by Steiger and that proposed by Steiger and Asmussen (vide supra) [48]. In addition, using measured moisture profiles, the fluid velocity can be calculated so that it is possible to determine what process, of advection or diffusivity, dominates the fluid transport.

### 4.2. Microscale Experiments

The aim of the microscale experiments was to study and determine the microscopic characteristics of salt crystallization and growth in both non-porous and porous media. These tests allow the researchers to assess (a) crystallization from a solution droplet at different relative humidity (RH) conditions and on different substrates; and (b) crystallization of salts in a stone sample. To this end, the so-called in situ techniques, such as environmental scanning electron microscopy (ESEM), X-ray diffraction (XRD), and atomic force microscopy (AFM), have been widely used (see Table 2). The ESEM is particularly advantageous vs. conventional SEM, since the samples can be studied in their natural state, even when they contain water. Furthermore, the possibility of controlling pressure (~2 - 20 Torr) and temperature (using a Peltier stage) in the ESEM chamber, and, in particular, near to the sample, enable condensation/evaporation cycles to be studied. Thus, high-magnification dynamic dissolution/precipitation studies of saline systems can be performed in situ in porous supports. Time-lapse digital images can be recorded online, and textural and/or morphological differences of hydrated salt crystals formed in the presence of additives can be observed, as well as real-time changes during crystallization or hydration/dehydration phenomena.

Ruiz-Agudo et al. [100,101], on the other hand, used in situ nanoscale observations of the {110} optically clear epsomite (MgSO_4_·7H_2_O) single crystals and of the {101̅4} calcite (CaCO_3_) surfaces during contact with saline solutions in the presence/absence of the additives by using atomic force microscopy (AFM). By using AFM, it was possible to observe the monomolecular layers and steps of the crystal surface in real time. In the first study by these researchers [100], the aim of the investigation was to evaluate the effects of several organic additives on the crystallization of epsomite, to have an indication of the possibility of their being used as a conservation treatment for salt-damaged ornamental stone. On the other hand, the study on Iceland Spar (calcite, CaCO_3_) single crystals [101] had the purpose of online monitoring of the crystallization of sodium and magnesium sulfates in the presence of DTPMP (diethylenetriaminepentakismethylphosphonic acid; C_9_H_28_N_3_O_15_P_5_), in order to gain a better understanding of the phosphonate-directed crystallization of inorganic salts onto calcite.

### 4.3. Molecular and Numerical Modeling

#### 4.3.1. Atomic-Scale Molecular Models

In other studies, computer simulations were employed to predict the crystal morphology, with particular reference to the presence of inhibitors.

Ruiz-Agudo et al. [51,100] used three different methods: (a) The Bravais, Friedel, Donnay, and Harker (BFDH) algorithm, which is an approximation based on a crystallographic geometrical calculation that uses the crystal symmetry and lattice to generate a list of possible growth faces and their relative growth rates. As a result, crystal morphology can be deduced. This approximated method does not take into account the energetics of the system. The method becomes less accurate when the bonding effects in the crystal are stronger. However, good approximations can usually be obtained, which makes this method useful for the identification of important faces in the growth process; (b) the attachment energy (AE) method, which can predict in a more accurate way the shape of the crystal, since the energetics of the system is taken into account. The so-called “attachment energy”, Eatt, is defined as “the energy release on the attachment of a growth slice to a growing crystal surface” [140]. The crystal face growth rate is proportional to its attachment energy, i.e., those faces exhibiting the lowest attachment energies are the slowest growing and, subsequently, have the greatest morphological importance. The attachment energy can be calculated for a series of suitable “slices” (hkl) that are chosen typically by carrying out a so-called Donnay–Harker prediction. From the energy calculation and, hence, the growth rate, a center-to-face distance is assigned to each face. This information can be used to deduce the morphology using a so-called Wulff plot [141]. Both BFDH and AE predict the relative growth rates of possible growth faces; and (c) on the other hand, the surface energy (SE) method predicts the equilibrium morphology of a crystal, which minimizes its total surface energy for a given volume and temperature [142]. If the surface energies are known for all relevant crystal faces, the morphology of a crystal in equilibrium with its surroundings can be visualized using the Wulff plot [141]. The surface energy is calculated from the energy of a specimen of finite thickness and is an average between the surfaces with Miller indices {h k l} and {-h-k-l}.

**Figure 10:**
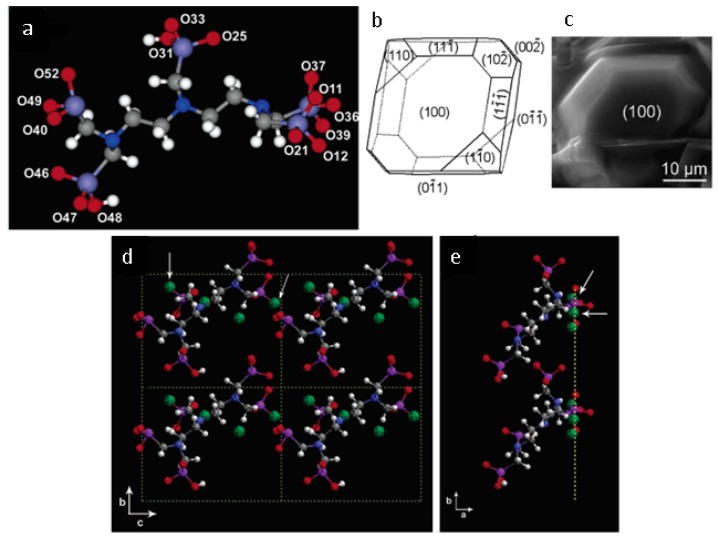
(**a**) Optimized molecular structures of DTPMP^8−^, i.e. DTPMP containing eight ionized polar groups [legend: (light blue) P; (red) O; (dark blue) N; (white) H; (gray) C]; (b-c) Morphology of mirabilite: (**b**) calculated (using the Bravais, Friedel, Donnay, and Harker – BFDH - method) and (**c**) experimental (environmental scanning electron microscopy photomicrograph). Note that the {100} faces are those with the greatest development; (**d**,**e**) Examples of possible docked positions of DTPMP^8-^ molecules on mirabilite (100) (four unit cells are represented), giving (**d**) top and (**e**) lateral views of the mirabilite (100) surface. For the sake of clarity, only Na cations of mirabilite have been represented. Arrows indicate bonding between Na and deprotonated functional groups of the phosphonate molecules [legend: (purple) P; (red) O; (dark blue) N; (white) H; (gray) C; (green) Na] [51,100]. Reproduced with permission from [51]. Copyright (2006) American Chemical Society.

Morphology calculations (BFDH, AE, and SE) were thus carried out in order to identify the key growth faces of the studied crystal, i.e., those faces that are more important from a morphological point of view since they exhibit slower growth rates, thereby being the most probable candidates for additive adsorption. The stereochemical matching of additives on crystal was modeled in several steps: (1) Surface cells were created from the mirabilite (Na_2_SO_4_·10H_2_O) unit cell at a given Miller plane (cleavage plane); this surface cell was further extended to a block of four cells; (2) spacing, e.g., for mirabilite, was measured between proximal Na atoms, H atoms, and sulfate groups, on different faces of the crystal; (3) oxygen–oxygen and phosphorous–phosphorous distances were determined between different deprotonated functional groups of additives. It is to be noted here that the structure of the most effective additive, in terms of inhibition (i.e. DTPMP), was previously optimized following the methodology outlined by Ruiz-Agudo et al. [51]; and (4) stereochemical matching on specific planes of mirabilite (*hkl*) was performed by substituting two sulfate groups with two phosphonate groups or by bonding two additive functional groups to (a) two Na atoms or (b) two H atoms (Figure 10).

This approach led to a good approximation as to whether the phosphonic acid groups in the additive would be capable of binding to at least two ions in the crystal lattice. It has been shown that for effective interaction between the additive and crystals, the molecule of the additive has to have several functional groups [143]. Note also that additives that bind simultaneously to two surface sites will be much more effective at blocking growth than a single ion molecule [144].

#### 4.3.2. Numerical Modeling

As mentioned above, the numerical simulation of different crystal growth processes at the micro scale, without considering the influence of the salt modifiers has been successfully implemented in the past. However, practically no reports are available in the literature, which deals with modeling salt crystallization phenomena in the presence of crystallization modifiers. More specifically, Kelnar et al. [122] developed a diffusion-advection model for salt solution transport, which considered both water movement due to the moisture gradient and diffusion and dispersion effects within the liquid phase due to the concentration gradient. Salt crystallization in the porous medium was modeled using an equilibrium model, taking into account the effect of crystallization inhibition. The computational simulations of three parameters, i.e., moisture diffusivity, salt ions’ diffusion coefficient, and moisture transfer coefficient, were realized using the so-called Galerkin finite element method. These three parameters were experimentally determined by carrying out a drying experiment of limestone samples saturated by sodium chloride (NaCl) solutions both with and without sodium ferrocyanide (Na_4_Fe(CN)_6_·10H_2_O; NaFe(CN)) inhibitor (0.001M). The drying experiments were performed using a NMR set-up developed by Gupta et al. [106] (see Section 4.1). In this apparatus, moisture and free chloride concentration profiles were measured every two hours. Kelnar et al. [122] demonstrated that in the process of drying of limestone saturated with NaCl solution, both water and salt ion (chloride) transport was slowed down by the presence of the inhibitor. This was ascribed to an increase of the viscosity of the salt solution and/or decrease of the interface tension in the pore walls. Nevertheless, the authors stated that this hypothesis needed to be verified by further experimental work.

More recently, Bracciale et al. [81] proposed a new mathematical model describing the effect of phosphocitrate (C_6_H_9_O_10_P; PC) on sodium sulfate (Na_2_SO_4_) crystallization inside a brick’s porous matrix through modelling of salt and water transport, and crystal formation in a 1-D symmetry. To this aim, two model parameters that are crucial for the appropriate description of an inhibitor were introduced: (a) The crystallization rate coefficient (K_s_), which depends on the nucleation rate, and (b) the specific volume of precipitated salt (γ), describing the crystal habit modification. These two parameters were determined by numerical fitting of the model for both the case of the brick treated with PC and untreated one. From the results of the fitting procedure of the mathematical model, the authors concluded that the presence of phosphocitrate (PC) increases the crystallization rate (K_s_ = 4.1·10^−5^s^−1^ and 6 ·10^−5^s^−1^ for untreated and PC treated, respectively) and decreases the crystal-specific volume (γ = 0.6 cm^3^g^−1^ and 0.53 cm^3^g^−1^ for untreated and PC treated, respectively). This means that, in the presence of PC, an increase in the nucleation rate occurs, although the crystals occupy a smaller volume, thus lowering the development of tensile stresses on the pore walls and ensuring hydraulic continuity into the porous matrix.

## 5. Conclusions

In this work, a survey of the most relevant publications on the use of chemical additives to mitigate and/or stop salt crystal growth in porous materials was given. The most representative inhibitors investigated so far belong to the class of alkaline ferrocyanides (K and Na ferrocyanides), phosphorous-containing species (phosphocitric acid and diethylenetriaminepentakis-(methylphosphonic acid)), and surfactants (sodium dodecylsulfate and ammonium cetyl dimethyl benzyl chloride). It was that salt crystallization inhibition processes in porous materials are dependent on a multitude of variables, including the porous substrate composition and properties, contaminant salt type/s and concentrations, microclimatic conditions, inhibiting solution concentration and properties, as well as application methods.

In the past decade, many of these often-interrelated factors and parameters in relation to the inhibition performance have been studied for different types of inhibitors. Salt crystallization tests, capillary rise tests, diverse instrumental techniques, and, more recently, computer simulation methods have been used over the time to assess qualitatively and quantitatively the effectiveness of the treatments.

It is now clear that impressive achievements have been obtained, leading in several cases to promising performances. Despite this, fundamental questions still need to be addressed, before crystallization inhibitors’ true potential in conserving building materials and built heritage can be assessed. Future developments should focus on further expanding the possibilities in terms of molecular structures; this should simultaneously involve:

- A careful design of targets, aided by “in silico” tools; tuning the inhibitor architecture in terms of, e.g., the molecular weight, nature and relative orientation of the functional groups, ionization degree, and charge distribution over the molecules, may enable an accurate control of the adsorption level/adsorption rate of the inhibitors to the crystal surface. On the other hand, it is mandatory to design architectures exhibiting low toxicity for humans and the environment, which will abide by relevant legislation applicable to the chemical substances. Additionally, it is desirable to take into account the stability and persistence in the environment of the inhibitors, as well as the toxicity of their environmental metabolites on soil microbial communities and on organisms of different levels of the trophic aquatic chain.
- The development of simple synthetic protocols (ideally employing biomass as a raw material), in which the use of expensive and toxic reagents as well as the waste associated with the chemical processes are minimized; these aspects will ultimately lead to efficient synthesis of the inhibitors at a scale much greater than that used in the laboratories.

## Figures and Tables

**Table 2:** Experimental method and techniques used.

Salt Type	Experiment Type	Material	Measurements	Ref.
CaSO_4_	Batch Crystallization Test	Salt Solution	Conductimetry; SEM	[70]
Na_2_SO_4_	Macroscale Crystallization Test	Monks Park Oolitic Limestone	ESEM; Evaporation Rate; MIP	[110]
	Batch Crystallization Test	Salt Solution	Conductimetry; Surface Tension; Contact Angle; Viscosity; Evaporation Rate; XRD; SEM-EDX	
			Conductimetry; Evaporation Rate; XRD; ESEM; Molecular Modelling	[51]
	Macroscale Crystallization Test	Brick; Tuff	Evaporation Rate; SEM	[49,75,78,80]
		Globigerina Limestone (*franka* (*bajda* and *safra*) and *soll*)		
	Microscale Crystallization Test	Glass frits	ESEM; 2D-XRD; TG-DSC	[137]
NaCl	Macroscale Crystallization Test	Limestone (Calcarenite from Spain)	SEM-EDX-CTL; XRD; AAS; MIP; Evaporation Rate	[103]
		Inhibitor addition Lime-Cement mortars	ESEM-EDX; *efflorescence* and Debris Content	[104]
	Batch Crystallization Test	Salt Solution	Conductimetry; Surface Tension; Evaporation Rate	[103]
	Drying Test	Salt Solution Droplets; Granada Limestone; Brick	NMR+Microscopy	[106]
NaCl+KCl; NaCl+LiCl		Salt Solution Droplets		[109]
NaCl; sea water	Macroscale Crystallization Test	Monçao and Rodas Granites	Evaporation rate; *efflorescence*/*subflorescence* Ratio; XRD; SEM	[105]
	Desalination Test		Conductimetry	
NaCl;Na_2_SO_4_	Macroscale Crystallization Test	Monks Park Limestone; Texas Cream Limestone	*efflorescence*/*subflorescence* Ratio	[102]
		Calcarenites from Noto and Palazzolo	Evaporation Rate; SEM-EDX	[77]
	Absorption-Drying Test; Macroscale Crystallization Test	Spanish Limestone; Czech Sandstone; Dutch Brick	ESEM-EDX; Evaporation Rate; HMC Content	[97]
NaCl; NaCl+Na_2_SO_4_	Macroscale Crystallization Test	Brick; Tuff	Evaporation Rate; SEM	[76]
MgSO_4_	Microscale Crystallization Test	{110} Optically Clear Epsomite Single Crystals	AFM	[100]
	Batch Crystallization Test	Salt Solution	Conductimetry; Evaporation Rate; XRD; ESEM; FTIR; Molecular Modeling	
Na_2_SO_4_; MgSO_4_	Macroscale Crystallization Test	Limestone (biocalcarenite from Granada)	ESEM; MIP	[138]
	Microscale Crystallization Test	{101̅4} Cleaved Iceland Spar Single Crystals; Salt Solution Droplets	ESEM; XRD; Morphology Simulation	
	Batch Crystallization Test	Salt Solution	XRD; Surface Tension; ESEM	
Na_2_SO_4_; MgSO_4_	Macroscale Crystallization Test	Granada Limestone	XRD; NMR; MIP	[102]
	Microscale Crystallization Test	{101̅4} Cleaved Iceland Spar Single Crystals	ESEM; AFM; RHXRD; Molecular Modelling	

SEM: Scanning Electron Microscopy; EDX: Energy-Dispersive X-ray Spectroscopy; ESEM: Environmental Scanning Electron Microscopy; MIP: Mercury Intrusion Porosimetry; XRD: X-Ray Diffraction; CTL: Cathodoluminescence; AAS: Atomic Absorption Spectroscopy; HMC: Hygroscopic Moisture Content; FTIR: Fourier Transform Infrared Spectroscopy; AFM: Atomic Force Microscopy; NMR: Nuclear Magnetic Resonance Spectroscopy; RHXRD: controlled RH/Temp. X-Ray Diffraction; TG: Thermogravimetric Analysis; DSC: Differential Scanning Calorimetry.
